# The functional significance of newly born neurons integrated into olfactory bulb circuits

**DOI:** 10.3389/fnins.2014.00121

**Published:** 2014-05-26

**Authors:** Masayuki Sakamoto, Ryoichiro Kageyama, Itaru Imayoshi

**Affiliations:** ^1^Institute for Virus Research, Kyoto UniversityKyoto, Japan; ^2^Kyoto University Graduate School of BiostudiesKyoto, Japan; ^3^World Premier International Research Initiative–Institute for Integrated Cell-Material Sciences, Kyoto UniversityKyoto, Japan; ^4^Japan Science and Technology Agency, Core Research for Evolutional Science and TechnologyKyoto, Japan; ^5^The Hakubi Center, Kyoto UniversityKyoto, Japan; ^6^Japan Science and Technology Agency, Precursory Research for Embryonic Science and TechnologyKyoto, Japan

**Keywords:** neurogenesis, main olfactory bulb, accessory olfactory bulb, granule cell, periglomerular cell, lateral inhibition, behavior, neural stem cell

## Abstract

The olfactory bulb (OB) is the first central processing center for olfactory information connecting with higher areas in the brain, and this neuronal circuitry mediates a variety of odor-evoked behavioral responses. In the adult mammalian brain, continuous neurogenesis occurs in two restricted regions, the subventricular zone (SVZ) of the lateral ventricle and the hippocampal dentate gyrus. New neurons born in the SVZ migrate through the rostral migratory stream and are integrated into the neuronal circuits of the OB throughout life. The significance of this continuous supply of new neurons in the OB has been implicated in plasticity and memory regulation. Two decades of huge investigation in adult neurogenesis revealed the biological importance of integration of new neurons into the olfactory circuits. In this review, we highlight the recent findings about the physiological functions of newly generated neurons in rodent OB circuits and then discuss the contribution of neurogenesis in the brain function. Finally, we introduce cutting edge technologies to monitor and manipulate the activity of new neurons.

## Introduction

It was believed that the adult mammalian brain is incapable of producing new neurons. The prominent histologist Cajal proclaimed “Once the development was ended, the founts of growth and regeneration of the axons and dendrites dried up irrevocably. In the adult centers, the nerve paths are something fixed, ended, and immutable. Everything may die, nothing may be regenerated.” (Ramon y Cajal, 1928). In 1960's, Altman and his colleague's pioneering study provided the first anatomical evidence of neurogenesis in the postnatal hippocampal region using a [H^3^]-thymidine incorporation labeling (Altman and Das, 1965). These [H^3^]-thymidine-labeling cells had neuronal morphology (Kaplan and Hinds, 1977). However, these findings were not accepted by Cajal's neuron doctrine that no new neurons are born in the adult brain. In 1980's, adult neurogenesis identified in songbird's brain was found to play a role in song learning (Goldman and Nottebohm, 1983). In 1990's, neural stem/progenitor cells were isolated from adult rodent brain, and adult neurogenesis was discovered in human hippocampus (Reynolds and Weiss, 1992; Eriksson et al., 1998). Since the discovery, adult neurogenesis has now become a well-accepted phenomenon including humans (Sanai et al., 2011; Bergmann et al., 2012; Spalding et al., 2013; Ernst et al., 2014).

In rodents, adult neurogenesis mainly occurs in two brain regions, the subventricular zone (SVZ) of the lateral ventricles and the subgranular zone (SGZ) of the hippocampal dentate gyrus (DG) (Kriegstein and Alvarez-Buylla, 2009; Suh et al., 2009; Aimone et al., 2011; Fuentealba et al., 2012). Adult neural stem/progenitor cells are regulated by many genes and signaling pathways (Kriegstein and Alvarez-Buylla, 2009; Suh et al., 2009). Neurons born in the SGZ migrate into the granule cell layer (GCL) and become granule cells of the DG, while neurons born in the SVZ migrate into the olfactory bulb (OB) through the rostral migratory stream (RMS), the pathway leading to the OB, and become local interneurons, granule cells (GCs) and periglomerular cells (PGCs) (Lledo et al., 2006; Ming and Song, 2011; Lepousez et al., 2013).

The olfactory system, which senses and processes odor information, is one of the oldest and important parts of the brain. Odor information is transferred to local neural circuits in the OB, and then conveyed to various regions of the olfactory cortex via principal neurons (mitral and tufted cells, hereafter referred to these neurons as M/T cells). Unlike most other central nervous system areas, GABAergic inhibitory interneurons greatly outnumber principal neurons, suggesting that odor representations in the OB are shaped by local inhibitory circuits (Yokoi et al., 1995; Isaacson and Strowbridge, 1998; Egger and Urban, 2006). Furthermore, although most neurons comprising the mammalian central nervous system are produced during embryonic development, a large proportion of these interneurons in the OB are generated and continuously renewed throughout life. Why do such continuous neuronal addition and replacement occur in the OB? Two decades of huge investigation revealed the biological importance of integration of new neurons into the olfactory circuits (Lledo et al., 2006; Kelsch et al., 2010; Ming and Song, 2011; Lepousez et al., 2013).

In this review, we highlight recent findings about physiological features of new neurons in rodent OB circuits and then describe the role of new neurons in olfaction-associated behaviors. Finally, we introduce optical techniques to monitor and manipulate the activity of new neurons.

### Neuronal circuit of the olfactory system

**Figure 1:**
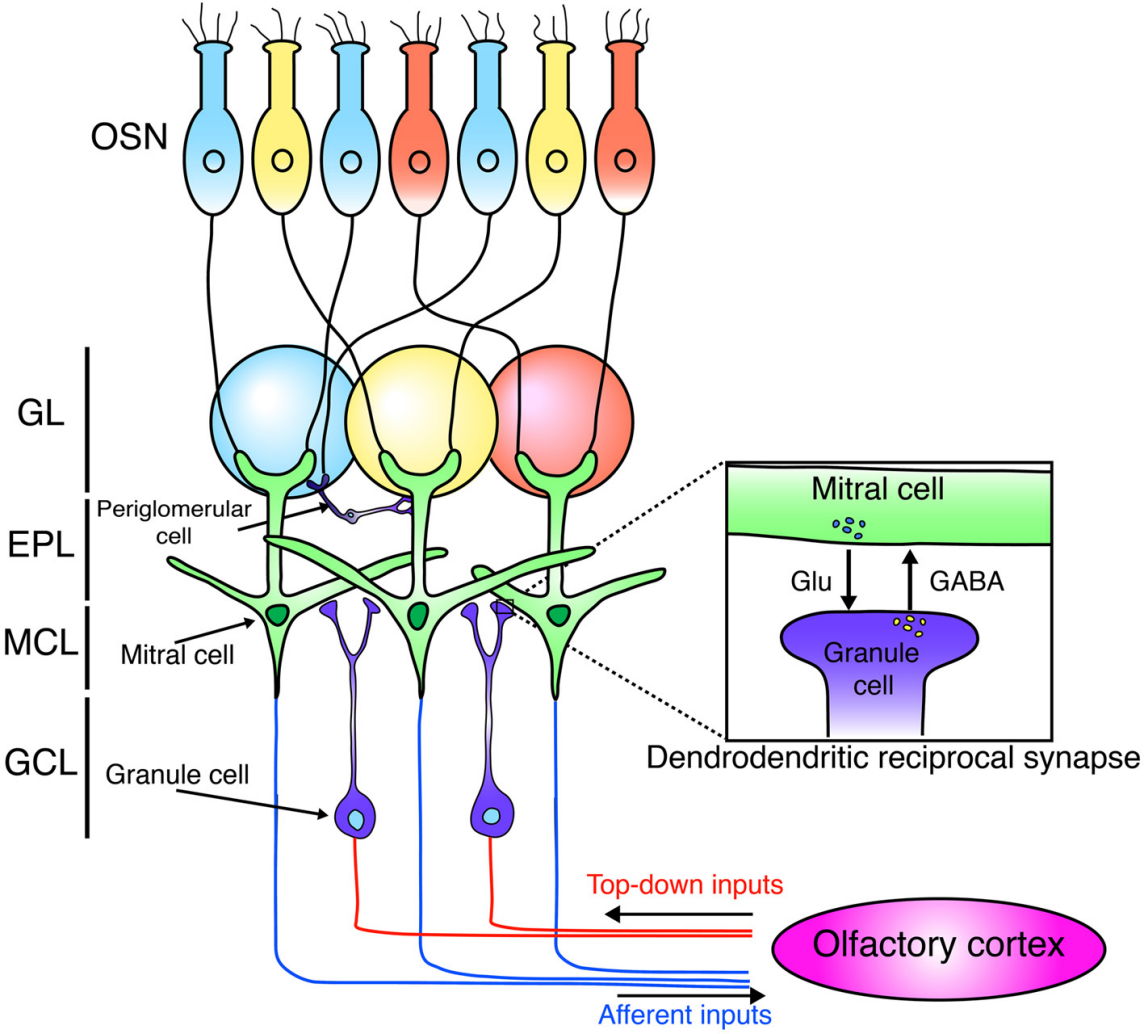
**Neural circuit of the olfactory bulb**. Schematic diagram of the neuronal circuit of the olfactory bulb. OSNs expressing the same odorant receptors (blue, yellow, red) project and converge their axons into the same glomeruli. OSNs form excitatory synapses with mitral cells. Mitral cells project their axons to the olfactory cortex. Mitral cells form dendrodendritic synapses with granule cells. Granule cells receive centrifugal glutamatergic inputs from the olfactory cortex. OSN, olfactory sensory neuron; GL, glomerular layer; EPL, external plexiform layer; MCL, mitral cell layer; GCL, granule cell layer.

The OB is the first relay station in the olfactory system that can process odor information (Figure 1). Odor information is detected by olfactory sensory neurons (OSNs). OSNs expressing the same odorant receptors project and converge their axons into the same glomeruli (Mori and Sakano, 2011). OSNs form excitatory synapses on primary dendrites of M/T cells. M/T cells project their axons to the olfactory cortex to covey odor information to higher brain areas in the forebrain. Mitral cells project their axons to nearly all areas of the olfactory cortex with a dispersed manner, while tufted cells target densely only to the anterior regions of the olfactory cortex (Ghosh et al., 2011; Miyamichi et al., 2011; Sosulski et al., 2011; Igarashi et al., 2012).

Synaptic connections in the external plexiform layer (EPL) of the OB are dominated by dendrodendritic reciprocal synapses between lateral dendrites of M/T cells and GCs, the latter being most numerous type of inhibitory interneurons in the OB. Unlike the neocortex, GABAergic inhibitory interneurons in the OB grealy outnumber principal neurons by 50–100:1 (Isaacson and Strowbridge, 1998; Egger and Urban, 2006). GCs form dendrodendritic synapses with M/T cells. In a dendrodendritic reciprocal synapse, both sides of the synapse are dendrites. M/T to GC is a glutamatergic excitatory synapse, while GC to M/T is a GABAergic inhibitory synapse (Figure 1). This large number of inhibitory synapses onto M/T cells may enable inhibitory circuits to refine odor representations. (Isaacson and Strowbridge, 1998; Egger and Urban, 2006; Lepousez and Lledo, 2013).

PGCs are another type of major GABAergic interneurons in the OB, and modulate the neural circuit in the glomerulus, consisting of terminals of the olfactory nerve and the dendrites of M/T. PGCs are subdivided into at least three subtypes based on immunoreactivity to calretinin (CalR), calbindin-28K (CalB), and tyrosine hydroxylase (TH) (Kosaka et al., 1998; Pressler and Strowbridge, 2006; Eyre et al., 2008, 2009; Kosaka and Kosaka, 2011). In mice, all three PGC subtypes seem to be GABA-expressing inhibitory neurons, but the definite functional roles of each PGC subtype in odor processing have not been well determined.

In addition to GCs and PGCs, numerous types of GABAergic interneurons have been identified in the OB (Pressler and Strowbridge, 2006; Batista-Brito et al., 2008; Eyre et al., 2008, 2009; Kosaka and Kosaka, 2011; Huang et al., 2013; Kato et al., 2013; Miyamichi et al., 2013), including deep short-axon cells, Blanes cells, and EPL interneurons. Although lineage and turnover analysis of these OB interneuronal populations has just been started (Batista-Brito et al., 2008; Bartolini et al., 2013), dynamic turnover of these interneurons by postnatal/adult neurogenesis may also contribute to the reorganization of OB circuitry.

### Unique features of newly generated neurons

While M/T cells are generated only at an embryonic stage, GCs and PGCs are generated throughout life (Imayoshi et al., 2008; Imamura et al., 2011; Sakamoto et al., 2014). Long-term genetic labeling analysis revealed that the majority of GCs are replaced by newly generated neurons during adult life (Imayoshi et al., 2008). Newly generated GCs are preferentially located in a deep region, while pre-existing GCs are located in a superficial region in the GCL of the OB (Lemasson et al., 2005; Imayoshi et al., 2008; Sakamoto et al., 2014). Interestingly, it has been shown that outer/superficial GCs, whose dendrites preferentially target the superficial lamina of the EPL, establish synapses with tufted cells, whereas deep GCs mainly contact the dendrites of mitral cells in the deep lamina of the EPL (Mori et al., 1983; Orona et al., 1983; Shepherd and Greer, 2004; Imamura et al., 2006). Therefore, one attractive hypotheis is that these two GC subpopulations fundamentally modulate distinct neural circuits. This implies that the activity of tufted cells is under the preferential control of embryonic-born GCs (static, superficial layers), while postnatal-born GCs (turnover, deep layers) provide an inhibitory drive to both mitral and tufted cells.

In rodents, although numerous new neurons reach the OB each day (roughly one percent of the total OB GCs), only half of them are integrated into pre-existing neural circuits. The remains of them are eliminated by apoptosis during their maturation (Lledo et al., 2006). This “survival or death” depends on olfactory sensory experience. Sensory deprivation triggers a decrease in new GC survival, whereas olfactory enrichment and learning boost the survival of these neurons (Petreanu and Alvarez-Buylla, 2002; Rochefort et al., 2002). Interestingly, day 14 to 28 after the generation is a critical period of newly born GCs when their survival is influenced by sensory experience (Yamaguchi and Mori, 2005). This time window overlaps with the period when newly generated neurons make synapses with pre-existing neurons, suggesting that synaptic inputs play a crucial role in the selection of adult born GCs (Kelsch et al., 2008; Yokoyama et al., 2011). Although the number of PGCs is one order smaller than that of GCs, new PGCs are also continuously produced throughout life (Ninkovic et al., 2007; Sakamoto et al., 2014). Like GCs, the survival of newly born PGCs is regulated in an activity-dependent manner. Sensory deprivation triggers a decrease in new PGCs' survival, whereas olfactory enrichment and learning boost the survival of adult generated PGCs (Rochefort et al., 2002; Alonso et al., 2006; Adam and Mizrahi, 2011; Sawada et al., 2011; Livneh and Mizrahi, 2012). A recent work also reported the generation of some glutamatergic short-axon cells at a very low proportion (Brill et al., 2009).

One recent elegant study provided direct evidence of the involvement of adult-born PGCs in olfactory sensory processing (Livneh et al., 2014). By using two-photon-targeted patch recordings, they showed that adult-born PGCs indeed respond to odor input. Interestingly, young adult-born neurons (2–4 weeks of age) have broader odor response profile than that of matured resident PGCs. Furthermore, sensory enrichment during developmental periods of adult-born neurons sharpens their odor response selectivity after maturation. These results indicated that continuous supply of these sensitive adult-born neurons into the olfactory circuit provides it with a mechanism of long-lasting plasticity (Livneh et al., 2014).

The OB receives input not only from OSNs but also from the olfactory cortex (Figure 1). This top-down input targets preferentially to the GCL and is important to shape the activity of M/T neurons (Manabe et al., 2011; Boyd et al., 2012; Markopoulos et al., 2012). In addition, recent studies showed that this cortical feedback is necessary for odor discrimination and food-intake (Nunez-Parra et al., 2013; Soria-Gomez et al., 2014). Furthermore, several studies showed the connectivity of newly generated neurons using monosynaptic rabies virus-based tracing system and revealed that newborn neurons in the OB receive glutamatergic inputs from neurons in the olfactory cortex (Arenkiel et al., 2011; Deshpande et al., 2013). Interestingly, new neurons exhibit more synaptic plasticity from centrifugal inputs than mature neurons do (Nissant et al., 2009). Furthermore, top-down inputs on the proximal dendrites of GCs also contribute to the survival/death of new neurons (Yamaguchi et al., 2013). Therefore, these observations imply that top-down glutamatergic input from the olfactory cortex to new GCs has a critical role in generating high plasticity in OB cirucuits.

Adult neurogenesis occurs in human brain as well as in rodents. Radiocarbon dating technologies revealed that adult neurogenesis in the OB is extremely limited though hippocampal neurogenesis occurs at a steady rate (Bergmann et al., 2012; Spalding et al., 2013). Surprisingly, new neurons born SVZ/lateral ventricles migrate and differentiate into striatum interneurons (Ernst et al., 2014). Furthermore, striatum neurogenesis is reduced in patients with Huntington's diseases. These results indicate that adult neurogenesis in humans has a unique pattern, and that these neurons derived from SVZ/lateral ventricles might be involved in brain functions such as cognition and motor coordination.

### The roles of newly generated neurons for olfaction-related behaviors

While the functional significance of continuous neurogenesis in hippocampus has been extensively studied (Deng et al., 2010; Aimone et al., 2011), the role of newly generated neurons in olfaction-related behaviors remains elusive. As mentioned above, newly born neurons form dendrodendritic synapses with M/T cells and control the activity of M/T cells to shape odor representation. Genetic ablation of newly born neurons in the OB impairs the structure and neural circuits in the OB (Imayoshi et al., 2008; Sakamoto et al., 2011). It was reported that newly generated GCs exhibit long-term synaptic plasticity, and that this ability is gradually lost as these neurons become mature, indicating that newly born GCs play a more important role in plastic change than mature GCs (Nissant et al., 2009). Importantly, electrophysiological recording revealed that ablation of adult born neurons impairs recurrent and lateral dendrodendritic inhibition of M/T cells and reduces the frequency of the induced gamma oscillations in the OB (Breton-Provencher et al., 2009). Furthermore, the survival of newly generated neurons is regulated by sensory experience (Yamaguchi and Mori, 2005; Lledo et al., 2006; Yokoyama et al., 2011). Together, these findings suggest that neurogenesis has a key role in olfaction-related plastic activities in the OB.

To understand the functional role of neurogenesis in the OB, various behavioral analyses have been applied. To address this question, various methodologies for inhibiting neurogenesis have been used: pharmacological, irradiation, and genetic targeting (Gheusi et al., 2000; Kim et al., 2007; Bath et al., 2008; Imayoshi et al., 2008; Breton-Provencher et al., 2009; Lazarini et al., 2009; Sultan et al., 2010; Sakamoto et al., 2011). Conversely, an apoptotic inhibitor was used to suppress cell death of newly born neurons (Mouret et al., 2009; Sultan et al., 2011).

**Figure 2:**
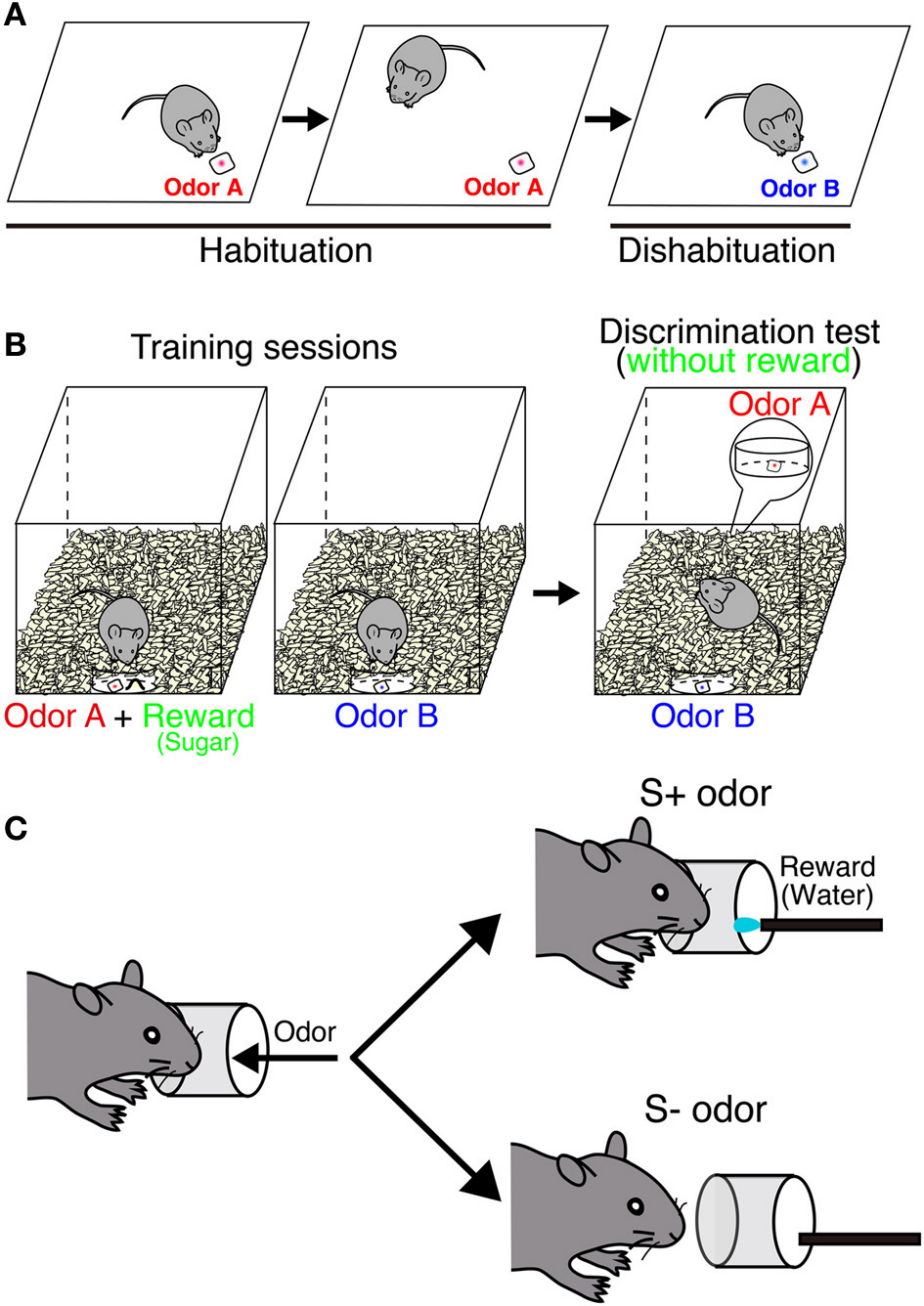
**Behavioral paradigm of odor discrimination test. (A)** Habituation-dishabituation test. In habituation sessions, odor A (red) is presented repeatedly. A mouse is habituated to this odor, and sniffing time is getting decreased. In a dishabituation session, sniffing time increases when the mouse can recognize a novel odor B (blue). **(B)** Odor-reward association test. During the training, a mouse is associated with one of the odors with reward (sugar). In the test, both odors are placed under the bedding separately without reward. Digging time of each odorant is measured to judge whether the mouse can discriminate between the two odors. In an odor memory retention test, mice are exposed to the same test without further training. **(C)** Go/no-go olfactory conditioning test. A mouse is associated with one of the odors with reward (water). Each trial is counted as correct if the mouse licks continuously upon presentation of a rewarded (S+) odor or does not lick continuously with a non-rewarded (S−) odor.

One of the simplest behavioral tests for olfaction is to check spontaneous odor exploration toward a novel odor without any rewards (Figure 2A). In this task, the ability of odor discrimination can be assessed by repeated presentations of the same odor (habituation) followed by the presentation of a novel odor (dishabituation). The sniffing time decreases during habituation sessions, but then increases when the odor is recognized as a new one in a dishabituation session. This can be applied to evaluate the odor detection threshold by comparing sniffing times between different concentrations. This test can also assess the short-term olfactory memory by changing time interval between sessions. Blockade of neurogenesis by infusion of an antimitotic drug impairs the ability of odor detection and short-term memory (60 min), suggesting that new neurons are involved in odor detection and processing odor memory (Breton-Provencher et al., 2009). In addition, gene deficient mice, which result in a decrease of new neurons in the OB, could not discriminate between dissimilar odors (Gheusi et al., 2000; Bath et al., 2008). However, not all findings have supported this result. Mice treated with γ-ray irradiation to block neurogenesis showed normal sensitivity (Lazarini et al., 2009), suggesting that spontaneous odor discrimination is not affected in these mice. Similar results were obtained from other studies (Kim et al., 2007; Imayoshi et al., 2008; Sakamoto et al., 2011). The discrepancies between these findings may be due to different ablation methods. Regarding the target specificity, blocking adult neurogenesis by using conventional knock in/out mice is far from specific.

Odor-reward association learning is another major paradigm that has been used to evaluate the function of neurogenesis in the OB (Figures 2B,C). This test can evaluate the capacity to associate an odor with reward (food or water) and the ability of odor discrimination and memory retention. Genetically ablated mice can acquire the odor-associated memory and maintain it for at least 2 months (Imayoshi et al., 2008; Sakamoto et al., 2011). Similar results were obtained from other animal models (Schellinck et al., 2004; Breton-Provencher et al., 2009). These results indicate that continuous neurogenesis is not required for simple discrimination between similar odors or retention of odor-associated memory. Pre-existing granule neurons born in embryonic and neonatal stages may compensate for these functions in the absence of new neurons. However, conflicting results were also reported following odor-reward association learning. Although mice showed normal odor discrimination ability, their long-term odor-associated memory retention was impaired by irradiation- or drug-induced inhibition of neurogenesis (Lazarini et al., 2009; Sultan et al., 2010). This discrepancy likely results from the difference in experimental paradigms. In addition, parameters to evaluate odor memory are totally different depending on behavior tests [digging time (seconds) or correct decision (%)].

These discrepancies may result from the target specificity for blockade of neurogenesis. Strictly speaking, these past methods did not specifically target new neurons in the OB. Neurons in other cortical regions might be affected to some extent. Therefore, there is a need to generate more sophisticated model that can target only newly generated neurons in the OB. One possibility to increase the target specificity is an intersectional strategy with dual site-specific recombinases (Cre and Flp) (Imayoshi et al., 2011; Huang and Zeng, 2013). Most of new neurons in the OB are GABAergic inhibitory neurons, while new neurons in the DG are glutamatergic excitatory neurons. Based on their different transmitter characteristics, new neurons in the OB and DG can be separately targeted. Recently, our group has developed new transgenic mice in which new neurons in the OB and DG can be separately targeted, and found that continuous neurogenesis is important for flexible olfactory associative learning and memory (Sakamoto et al., 2014). Continuous supply of new neurons in the OB is important to rewrite aquired odor memory and modify the value of odor-associated memory.

Adult neurogenesis in the hippocampus is required for pattern separation (Clelland et al., 2009; Sahay et al., 2011a,b; Nakashiba et al., 2012). It seems that odor enrichment improves a recruitment of newly born neurons and the olfactory discrimination ability. Blockade of neurogenesis with AraC impaired the improvement of odor discrimination, suggesting that neurogenesis is required for perceptual learning (Moreno et al., 2009). This result implies that neurogenesis in the OB also contributes to pattern separation. It would be interesting to examine the relationship between OB neurogenesis and pattern separation (Sahay et al., 2011b).

### Ob neurogenesis in innate behaviors

Adult neurogenesis is physiologically linked to reproductive behaviors, suggesting that continuous neurogenesis plays a pivotal role in pheromone-associated behaviors (Shingo et al., 2003; Mak et al., 2007; Mak and Weiss, 2010; Nunez-Parra et al., 2011). Pregnancy and lactation increase the number of both new GCs and PGCs (Shingo et al., 2003). Around gestation day 7, the proliferation reaches a peak in the SVZ/lateral ventricles. After the delivery, the number of new neurons integrated into OB circuits increases, and their dendritic spines exhibit stable features (Shingo et al., 2003; Kopel et al., 2012). These phenomena during early pregnancy and parenting might be important for fine-tuning of olfactory response to mating partners and pups. This induction of neurogenesis is mediated by prolactin (Shingo et al., 2003; Larsen and Grattan, 2010). Reducing the prolactin levels decreases neurogenesis in the SVZ/lateral ventricles and impairs maternal behaviors (Larsen and Grattan, 2010). Neurogenesis in females is also induced by pheromones of dominant males (but not other males) and is important for sexual behaviors (Mak et al., 2007; Oboti et al., 2009, 2011). Relevant increase of new neurons also occurs in male mice when they interact with their postnatal offspring (Mak and Weiss, 2010). This increase of neurogenesis mediated by prolactin appears to depend on the odor of their offspring and is involved in offspring recognition (Mak and Weiss, 2010).

These results indicate that OB neurogenesis is really related to sexual and maternal behaviors, suggesting that neurogenesis plays an important role in such pheromone-associated innately-programmed behaviors. Genetic inhibition of adult neurogenesis revealed that new neurons are essential for mating and maternal behaviors (Sakamoto et al., 2011). Blocking neurogenesis by injecting antimitotic drugs also impairs mating behaviors (Oboti et al., 2011). Pregnancy block (Bruce effect) is a well-known phenomenon; females terminate their pregnancy when they are exposed to the scent of unfamiliar males (Bruce, 1959). Although the detailed mechanism of this pregnancy block remains to be determined, it was shown that the pregnancy failure rate is highly increased by the blockade of continuous neurogenesis (Sakamoto et al., 2011). These results indicate that continuous neurogenesis is essential for pheromone-associated innately-programmed behaviors and activities. However, conflicting results were also reported. Disruption of neurogenesis in the OB by γ-ray irradiation left sexual and maternal behaviors unaffected (Feierstein et al., 2010). The discrepancy between these studies might derive from different models and target specificity as described above. One possibility is that newly born neurons in the DG might be involved in such behaviors. Moreover, because current available methods ablate new neurons in both the main and accessory olfactory bulb, it is difficult to conclude which is important for these pheromone-associated behaviors. More restricted ablating method will be required to address these questions.

Although the majority of newly born neurons are incorporated into the main olfactory bulb (MOB), a small number of new neurons migrate into the accessory olfactory bulb (AOB) (Oboti et al., 2009, 2011; Sakamoto et al., 2011). Genetic ablation of newly born neurons revealed that continuous neurogenesis is required for the maintenance of neuronal circuits in the AOB, as observed in the MOB (Sakamoto et al., 2011). However, unlike the MOB, adult neurogenesis does not lead to substantial replacement of GCs in the AOB. This result highlighted a unique integration mode of new neurons in the AOB, suggesting that intrinsic cellular and molecular properties of GCs may be different between the AOB and MOB. Further studies are necessary to elucidate cellular and molecular mechanisms underlying distinct features of GCs in the AOB.

### Optical imaging and manipulation of new neurons

Odor information processing is influenced by the activity of OB interneurons, including pre-existing neurons and newly born neurons. As mentioned above, neurogenesis contributes to various olfaction-related behaviors. However, how new neurons contribute to such behaviors is still unclear. New technologies are required to monitor and manipulate the activity of new neurons during such behaviors. Neuronal imaging technologies can help to tackle this issue. During the past decade, two-photon microscope has become a key tool for monitoring the structure, function, and plasticity of neurons *in vivo*. Calcium imaging is widely used to image the activity of many neurons simultaneously (Grienberger and Konnerth, 2012). In addition, two-photon calcium imaging can monitor the activity of OB GCs in the head-fixed awake state (Kato et al., 2012). However, calcium imaging operates too slowly to track the rapid firing of neurons and is also unable to measure the inhibitory signals. An alternative technique, voltage imaging, has a potential to overcome these problems and may ultimately enable to monitor spatiotemporal activity patterns with millisecond order (Peterka et al., 2011). Various kinds of genetically encoded voltage indicators have been developed (Knopfel, 2012). Recently, Akemann and his colleagues succeeded in voltage imaging at a single cell resolution with two-photon microscope *in vivo* (Akemann et al., 2013). It would be interesting to apply these technologies to examine physiological functions of newly born neurons in the OB. Because most OB newborn neurons are inhibitory interneurons, voltage imaging of M/T cells may help to clarify the contribution of GCs in olfactory circuitry more precisely than calcium imaging.

Optogenetics is also a powerful tool in the field of OB neurogenesis. Over the last decade, a wide variety of different kinds of opsins have been developed and become available, and now optogenetic approach is a standard methodology for investigating the functional properties of neurons at the circuit and behavioral level (Fenno et al., 2011). Recently, it was reported that the activation of newly born neurons by channelrhodopsin can accelerate difficult odor discrimination learning and improved odor-associated memory (Alonso et al., 2012). This strategy may also be useful to examine how newly born neurons contribute to pheromone-associated behaviors. Furthermore, optogenetic tools can control centrifugal input from the olfactory cortex to the OB (Boyd et al., 2012; Markopoulos et al., 2012). It will be interesting to examine how the top-down input affects the survival of new neurons and the effect of odor-associated learning (Yamaguchi et al., 2013). It would be also useful to express optogenetic probes in neurons under activity-dependent control. This approach can allow the reactivation or inactivation of only the subset of neurons that had been activated during a training phase and identify minimal ensemble that are required for behaviors. Light-reactivation of hippocampal neurons that are activated during the training can recall the fear memory of training task (Liu et al., 2012). Because new neurons express immediate-early genes in response to odor stimulation, this approach might be able to identify and manipulate newly born neurons that have been activated by odor stimulation (Magavi et al., 2005).

Although neurogenesis continues throughout life, newly generated neurons dramatically decrease in number with age, and this decline may be involved in memory deficit (Seki and Arai, 1995; Cameron and McKay, 1999; Encinas et al., 2011). In addition, aged mice are impaired at fine olfactory discrimination (Enwere et al., 2004). Furthermore, neurodegenerative diseases are relevant to OB function and adult neurogenesis. For instance, olfactory dysfunction is well known as an early symptom in Parkinson's disease although there is no specific change in the olfactory epithelium (Braak et al., 2003; Haehner et al., 2009). In Parkinson's disease model (α-synuclein overexpressing mice), the ability of odor discrimination is impaired and the survival of adult born neurons is reduced (Neuner et al., 2014). The next key challenge is to increase neurogenesis in aged/neurodegenerative brain and restore brain functions. Light-sensitive promoter system has a strong potential to achieve it (Wang et al., 2012; Imayoshi et al., 2013; Imayoshi and Kageyama, 2014). This system can control gene expression by blue-light illumination with reversibility. By applying this method *in vivo*, it might be possible to promote adult neurogenesis even in aged brains and lead to restore brain functions.

## Conclusion

Olfaction is indispensable in mammalian life. GCs are the most common GABAergic inhibitory neurons in the OB and modulate the activity of M/T cells to shape odor representations. The OB neural circuits are reorganized by incorporation and elimination of newly generated granule neurons throughout life. Furthermore, blockade of neurogenesis results in various olfaction-related behavior defects. Therefore, continuous neurogenesis is important to acquire plasticity in the olfactory system and thereby adapt neural circuits to environmental changes. However, there are still a lot of problems about adult neurogenesis to be solved. For example, molecular mechanisms integrating new neurons into the OB neural circuits is still unclear. In addition, there are some discrepancies about behavioral analyses. More sophisticated animal model and standardized behavior paradigms should be established. Further studies will contribute to solution of these problems and lead to the development of therapies and drugs for treatment of neurodegenerative diseases.

### Conflict of interest statement

The authors declare that the research was conducted in the absence of any commercial or financial relationships that could be construed as a potential conflict of interest.
